# Physical and Psychological Symptoms Associated With Premenstrual Syndrome and Their Impact on the Daily Routine of Women in a Low Socioeconomic Status Locality

**DOI:** 10.7759/cureus.10821

**Published:** 2020-10-06

**Authors:** Kiran Abbas, Ghazala Usman, Moiz Ahmed, Rabab Qazi, Ayesha Asghar, Aresha Masood Shah, Aliza Rizvi, Kanza Abid, Kousain U Haq, Amber Tahir, Syed Muhammad Usama

**Affiliations:** 1 Internal Medicine, Jinnah Postgraduate Medical Centre, Karachi, PAK; 2 Community Medicine, Jinnah Sindh Medical University, Karachi, PAK; 3 Internal Medicine, Jinnah Sindh Medical University, Karachi, PAK; 4 Internal Medicine, Dow University of Health Sciences, Karachi, PAK

**Keywords:** bloating, dysmenorrhea, irritability, menstruation, menstrual cycle, premenstrual syndrome, female reproductive health, quality of life, heath related quality of life, activities of daily life

## Abstract

Introduction

The constellation of the physical and psychological symptoms that appear several days before menstrual period is regarded as the premenstrual syndrome (PMS). The current study evaluated the symptoms associated with PMS and their impact on the day-to-day activities of women.

Methodology

An observational cross-sectional study was conducted at a squatter settlement in Karachi, Pakistan, from January 2019 to February 2020. Amenorrheic, pregnant women, and women who were on birth control at the time of data collection were excluded from the study. The demographics, symptoms of PMS experienced by the participants, and the impact of PMS symptoms on the daily lives of women were recorded. Statistical Package for the Social Sciences v.25 (IBM Corp., Armonk, NY) was used for data analysis.

Results

The mean age ± standard deviation of 23.93 years ± 9.41 years was recorded. As many as 213 (63%) women reported dysmenorrhea, followed by fatigue in 108 (32%), bloating in 64 (18.9%), and back pain in 45 (13.3%) women. Irritability and anxiety were experienced by 134 (39.6%) and 117 (34.6%) women, respectively. When asked about their attitude and perception towards menstruation, more than four-fifth respondents confessed that they feel impure when they are experiencing their monthly period. About 38 women (11.2%) believed that menstruation is God’s way of punishing the womankind. For the question, “Do you feel that your normal routine is significantly disturbed during your period?”, 40% responded in affirmation.

Conclusion

The findings of the current study reflected a generally negative attitude towards menstruation, which significantly affected the routine lives of women in our setting. The study further concluded that dysmenorrhea, fatigue, irritability, and anxiety were the most common symptoms of PMS experienced by women.

## Introduction

Menstruation is defined as a natural physiological process experienced by women of reproductive age, which involves monthly shedding and repairing of the uterine wall [1]. Menstruation initiates typically at the age of 11; the event is termed as “menarche” and continues until the cessation of fertility. Various physical and psychosocial aspects of menstruation adversely affect females, especially in developing countries [1,2].

In a recent study from Manipur, India, the prevalence of dysmenorrhea was reported to be 76%, and about 20% of participants experienced severe dysmenorrhea. The study claimed that due to the severity of these symptoms, the participants had to miss school that further deteriorated their academic performance, concentration in class and affected their social relationships and intervened with their daily physical activities [2].

The constellation of the physical and psychological symptoms that appear several days before the menstrual period is known as the premenstrual syndrome (PMS) [3]. Symptoms of PMS constitute backache, breast tenderness, abdominal bloating, weight changes, headache, mood swings, irritability, acne, stomach upset, cravings, or loss of appetite. Sharma et al. in their study reported frequency of dysmenorrhea, backache, fatigue, loss of appetite as 33%, 56%, 47%, and 28%, respectively, with almost 98.4% of the participants experiencing more than one symptom of PMS [4].

According to a study by Houston et al., PMS was the most common menstrual complaint (84.3%), including dysmenorrhea in 65%, abnormal cycle lengths in 13.2%, and excessive bleeding in 8.6% participants [5]. The women who experience these menses-related symptoms seldom receive the medical help they need as a result of which, their day-to-day activities are disrupted and cause discomfort, both physical and psychological [6]. The physical discomfort experienced during the menses is significantly related to psychosocial impairment. Titilayo et al. reported that participants who experienced dysmenorrhea had 1.5 times higher risk of suffering from depression compared to those women who did not suffer from PMS. The same study concluded that heavy menstrual bleeding affected the daily activities and social relationships among participants [7].

The physical and psychosocial experiences associated with menstruation inevitably affects the daily activities and quality of life of an individual. The sensitivity of the subject and the social taboo associated with menstruation in our society do not allow individuals to discuss the various attributes of this natural phenomenon openly. Therefore, a large proportion of young females remain uneducated and unaware about the many aspects of menstruation, hence making it impossible to improve their menstrual hygiene and overall quality of life [8].

While countless studies have addressed menstrual awareness and practices among women and young girls, there is a scarcity of data on the frequency and risk factors of PMS and how they impact women of reproductive age. The present study evaluated the frequency of both physical and psychological symptoms associated with PMS among women belonging to a shantytown in Karachi, Pakistan.

## Materials and methods

An observational cross-sectional study was conducted at the Bizerta Lines community (a low socioeconomic status locality with people from various social and cultural backgrounds), Karachi, Pakistan, between January 2019 and February 2020. Ethical approval was obtained from the Institutional Review Board (IRB), Jinnah Sindh Medical University (reference #JSMU/IRB/2020/-316). A total of 340 young girls and women participated in the study. Two of the participants left the study mid-way; they were excluded from the final analysis. A simple random technique was used to select households and schools using an electronic software, “random.org” (Randomness and Integrity Services Ltd., Dublin, Ireland) that creates truly random numbers using a computer algorithm. Once the households were selected, female members of those households who fulfilled the eligibility criteria were enrolled in the study. All menstruating women who had residence within the perimeter of Bizerta Lines, Karachi, and gave informed written consent were included in the study. Women who were amenorrhoeic, pregnant, menopausal, or had a history of birth control use were excluded from this study. The youngest participant of the study was 11 years old while the oldest was 47 years old.

The sample size was calculated using the Select statistics software (Select Statistical Services Ltd, Exeter, UK), keeping a confidence interval of 95%, margin of error as 5%, population of more than one million, and a 32.8% prevalence of PMS in the Pakistani population [9]. A sample size of 338 was obtained. After informed written consent was obtained, data were collected by trained researchers using a validated questionnaire. The pilot study was conducted on a demographically comparable subset of participants, and a Cronbach’s alpha was calculated to obtain a reliability of 0.85.

The questionnaire had three separate sections. The first section focused on the demographics of the participants, including age, marital status, employment or education status, ethnicity, and other information. The second section focused on the physical and psychological symptoms experienced by the participant just before or during menses and the impact they have on their daily lives. The last section discussed the general myths and misconceptions about menstruation. Statistical Package for the Social Sciences (SPSS) v.25 (IBM Corp., Armonk, NY) was used to analyze the data. Continuous data were reported as means plus standard deviations while the categorical data, including the frequency of symptoms related to premenstrual syndrome, were represented as a percentage. All data were further demonstrated in tabular or graphical forms.

## Results

A total of 338 women participated in the study. The mean age of respondents was 23.93 years ± 9.41 years, with an age range of 11 to 47 years. Half of the women were unmarried (165 [48.8%]). About 151 (15.1%) participants had attained education up to 10th grade, while 36 (10.7%) women had no formal education. About three-fifths of our study population was 11 to 17.9 years of age. Out of the two-fifth participants who aged between 18 and 47 years, more than 50% had employment (Table 1).

**Table 1:** Demographic characteristics of participants enrolled in the study (n=338)

Characteristics	Frequency n (%)
Age groups	
11-17.9 years	206 (61%)
18-47 years	132 (39%)
Marital status	
Unmarried	165 (48.8%)
Married	171 (50.6%)
Widowed	2 (0.6%)
Educational status	
No formal education	36 (10.7%)
Primary school	71 (21%)
Secondary school	160 (47.3%)
Higher secondary school or equivalent	151(15.1%)
Bachelor’s degree or equivalent	15 (4.4%)
Master’s degree or equivalent	5 (1.5%)
Employment status in the adult group	
Employed	77 (58.3%)
Unemployed	55 (41.6%)

On assessing the physical symptoms associated with PMS, the majority of the women (213 [63%]) reported abdominal cramping or dysmenorrhea, followed by fatigue in 108 (32%), bloating in 64 (18.9%), back pain in 45 (13.3%), breast tenderness in 40 (11.8%), acne in 29 (8.6%) and stomach upset in 29 (8.6%) women (Table 2).

**Table 2:** Physical and psychological symptoms experienced by the study participants during the menstrual period (n=338)

Symptoms	Age group	
	11-17.9 years	18-47 years
Physical symptoms		
Breast tenderness	22 (52.4%)	18 (42.8%)
Bloating	21 (32.8%)	43 (67.2%)
Abdominal pain	121 (56.8%)	92 (43.2%)
Back pain	8 (17.8%)	37 (82.2%)
Stomach upset	12 (41.4%)	17 (58.6%)
Fatigue	41 (38.0%)	67 (62.0%)
Acne	17 (58.6%)	12 (41.4%)
Psychological symptoms		
Irritability	59 (43.1%)	78 (56.9%)
Anxiety	63 (53.8%)	54 (46.2%)
Low mood	40 (51.3%)	38 (48.7%)
Stress	19 (31.1%)	42 (68.9%)
Hopelessness	8 (20.0%)	32 (80.0%)
Food cravings	13 (44.8%)	16 (55.2%)

Similarly, when psychological symptoms were assessed, it was found that the majority of the women experienced irritability and anxiety: in 137 (40.5%) and 117 (34.6%), respectively. Low mood, stress, and hopelessness were experienced by 78 (23.1%), 61 (18.0%), and 40 (11.8%) women, respectively. In 29 (8.5%) women, food cravings were also experienced just before or during the menstrual period. The frequency of symptoms associated with the premenstrual syndrome according to the age group of participants is demonstrated in Table 2.

When asked about their attitude and perception towards menstruation, more than four-fifth respondents confessed that they feel impure when they are experiencing their monthly period. Thirty-eight women (11.2%) believed that menstruation is God’s way of punishing the womankind. Nevertheless, more than three-fifths of the respondents thought of menstruation as a part of their healthy reproductive system. Most of the women experiencing the physical and psychological symptoms related to PMS reported a disturbance in their daily activities and routine during their menstrual period. For the question, “Do you feel that your normal routine is significantly disturbed during your period?”, two-fifth responded in affirmation. Only a minority, with a frequency of 54 (16%), claimed that they must take leave from school or work due to the severity of the physical or psychological symptoms associated with menstruation. The most common reasons for taking a leave from school or work was pain (in 32 [9.5%]), followed by a fear of staining, no access to a separate toilet room to change the soiled napkins, or even access to napkins. About 74 (21.9%) women claimed that they are unable to carry out their household duties/chores during their period while 64 (18.9%) claimed that they do not socialize as much as they would when they are not on their period (Table 3).

**Table 3:** Impact of symptoms of premenstrual syndrome on daily life activities (n=338)

Item	Frequency n (%)
Do you feel that your normal routine is significantly disturbed during your period?	
Yes	138 (40.8%)
No	200 (59.2%)
Do you ever take leave from school or work because of the symptoms you experience during your menstrual period?	
Yes	54 (16.0%)
No	284 (84.0%)
If you take leave from school or work during your period, state your reason?	
Afraid of staining clothes	14 (4.1%)
Pain/dysmenorrhea	32 (9.5%)
No place to wash/change/dispose of napkins	4 (1.2%)
Makes me feel uncomfortable	3 (0.9%)
Do not have access to sanitary napkins at school or workplace	1 (0.3%)
I do not take leave from school or work during my period	284 (84.0%)
Can you do household chores as per routine during your period?	
Yes	264 (78.1%)
No	74 (21.9%)
Do you socialize (go to family/friends’ gatherings, weddings, parties, etc.) during your period?	
Yes	274 (81.1%)
No	64 (18.9%)

## Discussion

The present study aimed to evaluate the frequency of physical and psychological symptoms experienced just before or during the menstruation and its impact on the daily activities of women belonging to a low-resource locality in Karachi, Pakistan. We also aimed to identify the attitude and beliefs of women regarding menstruation. It is well established that a lack of awareness and poor access to health-related education have been shown to promote the spread of inaccurate information regarding menstruation [10].

Dysmenorrhea was prevalent among most of the participants. A minority of participants who experienced the symptoms of PMS admitted to taking leave of absence from class or work, due to the severity of dysmenorrhea during or before menstruation. Since the participants belonged to a low socioeconomic level, many could not afford to take leave, especially when they were the sole breadwinners for their families. These findings are in line with a study reported by Omidvar et al. claiming that four-fifths of the study population suffered from primary dysmenorrhea that subsequently led to debilitating fatigue [11]. A systematic review of 38 studies concluded that dysmenorrhea/abdominal pain was frequently experienced by menstruating women and had a negative impact on their academic performance. The review claimed that 20.1% of women reported absenteeism from school or university due to unbearable pain or dysmenorrhea (N = 19, n = 11,226, 95% CI 14.9-26.7). Moreover, about two-fifths of women in these studies indicated that they experienced poor class performance and deteriorated concentration in class (N = 10, n = 5126, 95% CI 28.3-54.9) [12]. Most of the women in our study experienced disturbances in their routines.

Our study indicated that the majority of women experienced more physical symptoms in comparison to psychological distress during menstruation. For instance, the majority of the women, apart from experiencing dysmenorrhea, also complained of fatigue/tiredness and bloating or abdominal distention. In contrast, in an earlier study from Pakistan that assessed the impact of PMS on the overall quality of life (QoL) of medical students, Nusrat et al. reported that the majority of the women experienced psychological symptoms, including increased anger and irritability (83.8%). The most frequent physical symptoms experienced by the study population were breast tenderness, bloating, tiredness, among others, with a total frequency of 68.8%. Nevertheless, the study concluded that the symptoms of PMS negatively affected social life, activities, and the self-efficacy of the respondents [13].

The majority of the participants felt impure and unclean while menstruating, reflecting a generally negative attitude towards menstruation in our setting. The negative attitude towards menstruation can influence the overall experience of PMS. In a Turkish study on nursing students, the Menstrual Attitude Questionnaire (MAQ) was used to determine the general attitude of women towards menstruation and the Premenstrual Syndrome (PMS) Scale to assess its impact on the symptoms of PMS on women [14]. It was found that women with higher PMS symptoms scores also scored significantly higher on the debilitation portion of the MAQ scale while had substantially lower scores for the Denial subscale of the MAQ (p < 0.05). This reinforces the hypothesis that a negative attitude towards menstruation is linked with the increased frequency and severity of the symptoms of PMS experienced by women [15]. An earlier study explored the effects of the physical symptoms, like dysmenorrhea and heavy menstrual bleeding, on the health-related QoL (HRQoL) in a sample of 1575 Australian women aged 20 to 39 years [16]. The study concluded that the QoL was significantly impaired among women with severe PMS symptoms, further strengthening our findings.

Our study shows that PMS remains to be one of the most troubling experiences among women of reproductive age with a myriad of physical and psychological symptoms. A study by Rasheed and Al-Sowielem reported that out of the 464 study participants, 448 women (96.6%) experienced at least one PMS symptom with 176 (37.5%) women having severe symptoms [17]. Similar findings were reported by a study conducted in India with three-fifths (68.3%) of the female population claiming fatigue or lack of energy as the most frequent premenstrual symptom [18].

Despite many strong points, there were limitations observed in the study. The study was conducted in a specific locality of Karachi, Pakistan. Thereby, the data are generally applicable to those sharing similar demographic characteristics. Further studies should be undertaken to include a more diversified sample.

## Conclusions

PMS remains one of the most troubling disorders women of reproductive age experience, with a constellation of distressing symptoms. The present study concluded that dysmenorrhea, fatigue, irritability, and anxiety were the most common physical and psychological symptoms of women's PMS in our setting. It negatively affects the routine lives of women compromising their academic and professional efficiency. Almost one-fourth of the study population believed their periods to be nature's punishment for women; with attitudes like these, a majority of adolescent girls are missing out on the opportunity to receive health education on the diagnosis and treatment of their menstrual problems. Relaxing exercises, proper medical attention, relief from work, and female-oriented therapy may lessen or improve PMS symptoms. This study paves the way towards dispelling negative attitudes towards menstruation.
